# FLINT INTERGENERATIONAL LIVING EXPERIENCE

**DOI:** 10.1093/geroni/igac059.1973

**Published:** 2022-12-20

**Authors:** Jennifer Blackwood, Elizabeth Yost

**Affiliations:** University of Michigan-Flint, Flint, Michigan, United States; University of Michigan-Flint, Flint, Michigan, United States

## Abstract

Previous studies indicate that students who have a formative experience with an older adult, in a personal or professional setting, are more likely to choose to work in geriatrics. With a growing population of older adults, the need for empathetic health professionals who desire to work in geriatrics is needed. In Fall, 2021 four graduate students (2 physical therapy, 2 occupational therapy) moved into a senior housing community in Flint, Michigan. From that moment, the Flint Intergenerational Living Experience (FILE) began. The purpose of FILE is to mold these students into empathetic future health professionals who choose to work in geriatrics within their careers. These four twenty-something year-olds live, learn, study, eat, and share life with their senior neighbors with the hope of gaining a better understanding of aging issues of older adults who chose to remain in Flint. In exchange for completing service hours, the FILE students live in an apartment complex rent-free. This presentation will describe the activities performed, outcomes achieved, and perceptions of the FILE program after one-year of being in the program. Based on data gathered within the first year of the program, activities that impacted the personal growth of the students to understand the impact of aging in an urban setting will be described. The influence of loss (e.g. death, physical abilities) and the framework of rehabilitation needs of the urban dwelling older adults will be discussed. Lastly, strengths and weaknesses of this lived experience will be presented.

